# Elucidating the Multi-Target Anti-Pruritic Mechanism of *Polygonatum odoratum* via Integrated Network Pharmacology, Molecular Simulations, and GEO Dataset Validation

**DOI:** 10.3390/cimb48040369

**Published:** 2026-04-01

**Authors:** Jiabei Chen, Chenglu Liu, Xinbo Chen, Guoliang Yu, Zhen Li, Hua Yang

**Affiliations:** 1 College of Bioscience and Biotechnology, Hunan Agricultural University, Changsha 410128, China; chenjiabei2023@163.com (J.C.); r3654236@163.com (C.L.); chenxinbo@hunau.edu.cn (X.C.); lizhen_905@163.com (Z.L.); 2 Yuelushan Laboratory, Changsha 410128, China; yuguoliang2025@163.com

**Keywords:** pruritus, *Polygonatum odoratum*, network pharmacology, molecular docking, molecular dynamics simulations, levocetirizine

## Abstract

*Polygonatum odoratum*, a medicinal and edible plant widely used in traditional Chinese medicine and daily diets, has potential in managing various disorders, but its anti-pruritic mechanisms remain unclear. This study aimed to explore its multi-target anti-pruritic effects by integrating network pharmacology, molecular docking, molecular dynamics (MD) simulations, GeneMANIA functional association analysis (GMFA), and GEO dataset validation. Bioactive components and pruritus-related targets were identified from public databases, and interaction networks between *Polygonatum odoratum* and pruritus targets, as well as the antihistamine levocetirizine, were constructed. Core targets were screened, and functional enrichment analyses were performed using DAVID and KEGG. Molecular docking (AutoDock Vina) and MD simulations (AMBER20) assessed the binding energy and stability of core components with key targets. The analysis identified 5 active components, 208 related targets, and 113 pruritus-associated targets, including 10 core targets. Enrichment analysis highlighted the PI3K/Akt and IL-17 signaling pathways, while MCODE clustering suggested involvement in arachidonic acid metabolism and serotonergic synapse. GMFA supported these findings. Molecular docking showed strong binding energy (<−5 kcal/mol), and MD simulations confirmed stable ligand–target complexes. GEO dataset validation reinforced key results. This study suggests that *Polygonatum odoratum* may exert anti-pruritic effects through the combined actions of inflammation suppression, skin barrier repair, and neural modulation, revealing a novel multi-target mechanism for pruritus therapy and potential synergy with levocetirizine.

## 1. Introduction

Pruritus, commonly referred to as scratching or pruritus, is an exceedingly prevalent clinical symptom that can arise from a diverse array of dermatological conditions and systemic diseases. Conditions such as atopic dermatitis, psoriasis, contact dermatitis, urticaria, lichen simplex chronicus (also known as neurodermatitis), mycosis fungoides, cicatricial disorders, autoimmune diseases, and renal or hepatic pathologies can induce pruritus, thereby significantly impairing the quality of life of affected individuals [1,2]. In contemporary clinical practice, antihistamines and corticosteroids are predominantly employed as first-line therapeutic agents. However, antihistamines often exhibit limited efficacy in treating chronic pruritus or pruritus that is not mediated by histamine release. Moreover, they are associated with a range of adverse effects, including somnolence, xerostomia, dry eyes, and urinary retention [3,4]. Additionally, long-term or excessive use of topical corticosteroids may lead to side effects such as skin thinning, skin pigmentation, and increased susceptibility to bleeding [5]. Therefore, for chronic, refractory, or non-histaminergic pruritus, existing treatment options remain significantly limited, highlighting an urgent need to explore novel therapeutic strategies that are safe, effective, and backed by well-defined mechanisms. Therefore, the identification of safe, highly targeted natural bioactive compounds to complement existing therapeutic approaches holds significant clinical value.

*Polygonatum odoratum* (Mill.) Druce (*P. odoratum*), a perennial herb belonging to the genus *Polygonatum* (family Asparagaceae), serves as both a medicinal herb and edible plant, and is widely distributed across East Asia and Europe [6]. It has been used as a traditional Chinese medicine since ancient times without toxic or side effects, and is also consumed as a vegetable, food ingredient, or herbal tea [7]. At present, various bioactive phytochemicals have been isolated from *P. odoratum*, including polysaccharides, flavonoids, glycosides, saponins, and alkaloids [8]. *P. odoratum* exhibits diverse pharmacological activities, including preventing and treating hyperlipidemia, hyperglycemia, obesity, and cardiovascular diseases, as well as exerting antioxidant and immunomodulatory effects [9]. These activities align with the core pathogenesis of pruritus. The occurrence of pruritus is often associated with the release of inflammatory factors, elevated oxidative stress, and immune imbalance, while the anti-inflammatory, antioxidant, and immunomodulatory effects of *P. odoratum* target precisely these pathological links. However, despite the multiple health benefits demonstrated by Solomon’s seal in both traditional and modern applications, its specific molecular mechanisms and potential targets in anti-itching—particularly in chronic or inflammation-related pruritus—remain poorly understood. Systematic research in this area is lacking, and the underlying pharmacological actions require further elucidation.

Network pharmacology, a systems biology approach pioneered by Hopkins in 2007 [10], provides a powerful framework for investigating multi-target drug mechanisms [11,12,13,14,15]. This methodology is particularly suited for elucidating the complex molecular interactions underlying chronic inflammatory conditions. Additionally, molecular docking is a computational technique that simulates molecular structures and interactions to identify the best binding mode between small-molecule drugs and larger biomolecules. Various docking methods exist, though some have limitations, like simplified scoring functions. Molecular dynamics simulations can provide atomic-level insights into the relationship between biological processes and function, helping to clarify biological mechanisms [16]. Molecular dynamics simulations may also compensate for certain limitations and activate otherwise inactive molecules [17]. Therefore, the integration of methods such as network pharmacology, molecular docking, molecular dynamics simulations, and functional network analysis enables efficient elucidation of interactions between chemical substances and biological targets, providing a high-throughput research pathway for revealing the complex mechanisms of action in traditional Chinese medicine.

This study takes *P. odoratum* as the core research object, focusing on the potential targets of its bioactive components in treating pruritus and exploring the underlying molecular mechanisms. A combination of multiple technical approaches was employed herein, including network pharmacology, molecular docking, molecular dynamics simulation, GeneMANIA-based functional association (GMFA) analysis, and GEO dataset validation. Specifically, network pharmacology was used to screen the potential targets and related pathways of bioactive components in *P. odoratum* and construct the “component-target-pathway” network; molecular docking and molecular dynamics simulation were adopted to verify the binding activity and stability between core bioactive components and targets; GeneMANIA-based functional association analysis was utilized to explore the functional network correlations among hub targets, further clarifying the role of *P. odoratum* bioactive components in regulating pruritus-related physiological and pathological processes; GEO dataset validation was performed to ensure the reliability and clinical relevance of key findings. Collectively, this study identifies novel potential drug candidates for pruritus treatment, opens up new directions and perspectives for future research on *P. odoratum*, and provides fresh insights and strategies for its clinical application.

## 2. Materials and Methods

### 2.1. Screening and Validation of Active Compounds Based on Drug-Like Properties and Target Association

The Traditional Chinese Medicine Systems Pharmacology Database and Analysis Platform [18] (TCMSP, https://www.tcmsp-e.com/load_intro.php?id=43, accessed on 27 July 2024) was utilized to find the compounds in *P. odoratum*. The main components were screened based on oral bioavailability (OB) ≥ 30% and drug likeness (DL) ≥ 0.18. The PubChem [19] (https://pubchem.ncbi.nlm.nih.gov/, accessed on 27 July 2024) database was accessed to retrieve the compounds in canonical SMILES format. The pharmacokinetic and ADME profiles of the screened compounds, along with their canonical SMILES notation, were assessed using SwissADME (http://www.swissadme.ch/, accessed on 5 April 2025) [20]. The screening criteria were as follows: high gastrointestinal absorption, compliance with the five drug-likeness rules (Lipinski, Ghose, Veber, Egan, Muegge), and oral bioavailability > 0.5 [21,22]. SwissTargetPrediction [23] (http://swisstargetprediction.ch/, accessed on 28 July 2024) was employed to identify the potential targets of the active compounds of *P. odoratum*, with the search limited to human targets (Homo sapiens) and probability ≥ 0.1 [24,25,26]. The predicted targets were supplemented with known targets of the active compounds from the published literature. Subsequently, the target names were converted to standard gene names via the UniProt database [27] (https://www.uniprot.org/, accessed on 5 August 2024).

### 2.2. Pruritus-Related Targets

The keyword “PRURITUS” was used to search for targets related to pruritus in the TTD (https://db.idrblab.net/ttd/, accessed on 28 July 2024) [28], DrugBank (https://go.drugbank.com/, accessed on 28 July 2024) [29], and Human GeneCards (https://www.genecards.org/, accessed on 28 July 2024) [30]. For the search in GeneCards, the relevance score was set to be greater than the median of 0.3667 as the screening criterion.

### 2.3. Selection of Standardized Drugs with Pruritus as the Main Symptom and Collection of Relevant Targets

Levocetirizine is a third-generation antiallergic drug that belongs to the class of antihistamines. As the levorotatory enantiomer of cetirizine, levocetirizine exhibits pharmacologic effects similar to those of cetirizine but with a reduced incidence of adverse effects [31,32]. In clinical trials, levocetirizine has demonstrated significantly greater efficacy in treating symptomatic chronic idiopathic urticaria compared with the second-generation antihistamine desloratadine [33]. To further elucidate the mechanism of action of levocetirizine, a search on GeneCards was conducted to identify its potential molecular targets.

### 2.4. Construction of Protein–Protein Interaction (PPI) Networks

To elucidate the relationship between pruritus and the genes associated with *P. odoratum* and levocetirizine—a well-known therapeutic agent for pruritus—we employed the Venn Diagrams online tool v2.1.0 [34] (https://bioinfogp.cnb.csic.es/tools/venny/index.html, accessed on 26 February 2025) to identify the intersection of active compounds of *P. odoratum*, levocetirizine, and pruritus-related proteins. The STRING database (https://string-db.org/, accessed on 3 July 2025) [35] was utilized to construct protein–protein interaction (PPI) networks for the common targets of *P. odoratum* active compounds and pruritus, as well as for levocetirizine and pruritus, respectively. The target organism was set to Homo sapiens, with a confidence score threshold of 0.9. Disconnected nodes in the networks were removed to enhance clarity. The PPI networks for *P. odoratum* active compounds and pruritus were visualized using Cytoscape v3.10.0 software, and the topological parameters of these networks were analyzed using Cytoscape’s network analyzer tool. The CytoNCA plugin was employed to identify core targets of *P. odoratum*’s action on pruritus, with a threshold value set greater than the median of the median centroid, tight centroid, and degree. Additionally, gene combinations that were more tightly linked within the *P. odoratum* active compounds and pruritus PPI network were analyzed using the MCODEv2.0.3, a plugin for Cytoscape, with parameters set to default.

### 2.5. GO and KEGG Enrichment Analysis

The overlapping targets of *P. odoratum* with pruritus and levocetirizine were uploaded to the DAVID database [36] (https://davidbioinformatics.nih.gov/, accessed on 3 July 2025). The analysis was conducted with the species set to Homo sapiens and a significance level of *p* < 0.05. The *p* values were sorted in ascending order. For the Gene Ontology (GO) analysis, the top 10 terms across biological process (BP), cellular component (CC), and molecular function (MF) categories were selected, along with the top 20 KEGG pathways for visualization. Additionally, enrichment maps of key GO terms and pathways were generated using a tool on the SRplot web server [37] (https://www.bioinformatics.com.cn/srplot, accessed on 2 September 2025).

### 2.6. Drug–Component–Target–Pathway Network Construction

To elucidate the anti-pruritic mechanism of *P. odoratum*, a drug–component–target–pathway network was constructed using Cytoscape v3.10.0. Within this network, distinct nodes represented drugs, compounds, targets, and target-related pathways, each distinguished by unique colors and shapes.

### 2.7. Molecular Docking Validation

To explore the binding interactions between core targets and key components of the traditional Chinese medicine *P. odoratum*, molecular docking simulations were conducted. In this study, we adhered as closely as possible to the “high-resolution, human-origin, co-crystallized ligand” priority principle when selecting protein structures from the RCSB PDB database [38] (https://www.rcsb.org/, accessed on 7 September 2025). Although resolution is an important quality indicator, molecular docking places greater emphasis on the functional relevance of the active site conformation, necessitating a balanced consideration. For instance, although AKT2 (PDB ID: 3e8d, 2.70 Å) is not of the highest resolution, it was co-crystallized with an ATP-competitive inhibitor and a substrate peptide, thereby retaining the active kinase conformation, which is more valuable for inhibitor docking studies. The selection of JUN (PDB ID: 1s9k, 3.10 Å) was constrained by the availability of a human-origin structure with a complete binding site. The selection of these and other protein structures is supported by the literature [39,40,41,42,43,44,45,46,47,48]. For targets classified as transcription factors, this study investigates the potential mechanisms underlying the modulation of their transcriptional activity by exploring the binding of small-molecule ligands to functional interfaces of transcription factors (including AP-1, etc.)—such as DNA-binding surfaces and protein–protein interaction interfaces. Therefore, docking analyses are not limited to classical ligand-binding pockets but also include scanning of these functional surfaces. Preprocessed using PyMOL 2.3.0. The key components were preprocessed using Chem3D 20.0. Subsequently, these preprocessed components were docked with the lattice files generated from the preprocessed PDB proteins using AutoDock Vina 1.1.2. We first docked each ligand to its target with AutoDock Vina and kept the lowest-energy pose. The docking grid was configured using the protein active-center expansion approach. With the centroid of the co-crystallized ligand or the center of the functionally reported pocket as the reference, a cubic grid box (GridBox) was established. The specific parameters of the GridBox for each protein are provided in Supplementary Table S1. This pose was then visualised in PyMOL 2.3.0 and Discovery Studio 2019, and its noncovalent contacts were analysed with the online protein–ligand interaction profiler [49] (https://plip-tool.biotec.tu-dresden.de/plip-web/plip/index, accessed on 21 December 2025). A heat map summarising the docking scores was prepared with TBtools-II v 2. 112.

### 2.8. Molecular Dynamics Simulation

Following molecular docking using AutoDock Vina software, complexes with low binding energies were prioritised from the docking results. After verifying structural validity using PyMOL, complexes with both low binding energies and structural stability were ultimately selected for molecular dynamics simulations. The molecular dynamics simulation was performed using AMBER20 software, with the Gaff2 force field applied. For protein-related simulations, the ff14SB force field was adopted, and the explicit solvent tip4pew force field was utilized for solvation. Small molecule RESP charges were computed by the BCC method. Topology (.top) and coordinate (.crd) files for all systems were generated through TLEAP. The simulations were executed under pressure-free conditions (ntp = 0 and ntb = 1), initiated with heating to 300 K (starting temperature set at 300 K, with tempi = 100 and temp0 = 300). In the subsequent kinetic simulations, amino acid residues of experimentally crystallized proteins were initially constrained and pre-optimized for 5 ns to guarantee proper hydrogenation and substrate alignment. After that, the constraints were released, and molecular dynamics simulations continued for another 100 ns. RMSD and associated analyses were carried out on trajectory files using cpptraj (as implemented in AMBER20). The heating phase of the simulation was performed under the NVT ensemble (ntp = 0, ntb = 1) to ensure a smooth temperature transition; the subsequent 100 ns production simulation, regarded as the final simulation stage, was carried out under the NPT ensemble (ntp = 1, ntb = 2) to analyze the structural stability of the complexes. Additionally, the binding free energies reported here were calculated by the MM-PBSA method. All molecular structures were visualized and graphically represented using PyMOL v2.3.0.

### 2.9. GeneMANIA-Based Functional Association Network of Core Targets and Extended Database Analysis of Potential Therapeutic Targets

In this study, GeneMANIA [50] (https://genemania.org/, accessed on 8 July 2025) analysis was used to assemble a comprehensive set of genes with newly identified functional correlates for the top 10 core targets of *P. odoratum* in pruritus management. The analysis centered on three key aspects: co-expression, genetic interactions, and physical interactions. For each core target, the top 20 additional genes with the strongest correlations were identified and prioritized within the gene-gene network. This approach aimed to capture critical genes involved in disease processes and to identify a broader range of related genes [51,52]. Subsequently, the top 10 newly identified genes per core target were merged with the initial 10 core targets to construct an extended GMFA-extended database (GMFA-ED) for pruritus-targeted *P. odoratum* research.

We employed DAVID (https://davidbioinformatics.nih.gov/, accessed on 21 August 2025) to carry out GMFA-ED based GO and KEGG pathway enrichment analyses, focusing on the KEGG results. For the PI3K-Akt pathway targets identified in the KEGG set analysis of GMFA-ED, we retrieved the corresponding protein structures from the RCSB PDB database and conducted molecular docking and MD simulations with the key components of *P. odoratum*, following the previously described steps.

### 2.10. Differential Expression Analysis of Core Targets for P. odoratum Treatment of Itching Based on GEO Database Validation

Using the GEO2R online tool, we analyzed the itch-related dataset GSE6281 from the NCBI GEO database [53] (https://www.ncbi.nlm.nih.gov/geo/, accessed on 8 September 2025) (Platform: GPL570, [HG-U133_Plus_2] Affymetrix Human Genome U133 Plus 2.0 Array, nickel-allergic patients after nickel stimulation (96 h) vs. nickel-allergic patients before nickel stimulation (0 h): 6 vs. 4), GSE76446 (Platform: GPL570, same as above, formaldehyde-exposed human primary keratinocytes vs. normal human primary keratinocytes: 2 vs. 2). These two models represent typical pathological pruritus induced by allergens and irritants, which fit well with the inflammatory and immune mechanisms of pruritus in this study. Due to the independent study designs and differing platforms, differential analysis was performed separately for each dataset without merging. All preprocessing employed GEO2R default RMA background correction and quantile normalization; probes with “///” multiple annotations were manually excluded. Differential expression was defined by |log_2_FC| > 1 and *p* < 0.05. Volcano plots are generated using the built-in tools on the SRplot web server [37]. Venn diagrams were employed to identify core targets of *P. odoratum*’s effects on pruritus and their overlapping action targets with both pruritus-related disease datasets.

## 3. Results

### 3.1. Intersecting Targets Between P. odoratum, Levocetirizine, and Pruritic Targets

**Figure 1:**
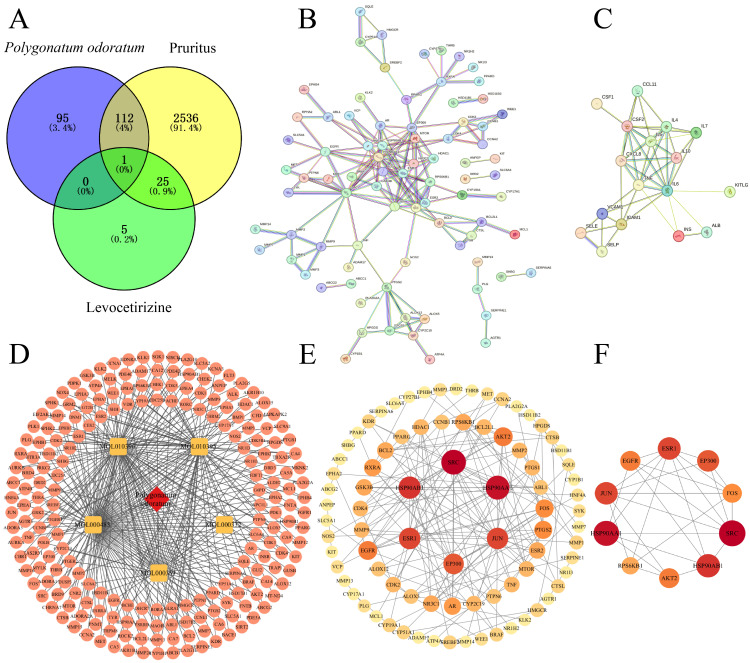
Targets at the intersection of active ingredients and disease. (**A**) Venn diagrams of the therapeutic targets of *P. odoratum*, the therapeutic targets of levocetirizine, and the pruritus-related targets. (**B**) PPI network of the active compounds of *P. odoratum* and the potential targets of pruritus. (**C**) PPI network of levocetirizine and the potential targets of pruritus. (**D**) “Traditional Chinese Medicine-Active Ingredient-Target” network. (**E**) Cytoscape software after processing the B diagram. (**F**) Core target regulatory network filtered from the E diagram.

Nine active ingredients of *P. odoratum* were screened according to the drug-like principle of OB (oral bioavailability) ≥ 30% and DL (drug-likeness) ≥ 0.18, as shown in Table 1. Only five ingredients, MOL000332, MOL010396, MOL010395, MOL000483, and MOL000359, had corresponding targets, and 208 drug targets were selected in total (Supplementary Table S2). In addition, the five ingredients’ SwissADME druggability scores were consistent with Lipinski’s Rule of Five and oral bioavailability > 0.5. Levocetirizine collected 31 drug targets. By organizing the targets obtained from TTD, Drugbank, and GeneCards, 2674 pruritus-related targets were identified. In the Venn diagram, there was only 1 common target of TNF for scratching, *P. odoratum* active ingredient, and levocetirizine, 113 intersecting targets of *P. odoratum* active ingredient with pruritus, and 26 intersecting targets of levocetirizine with pruritus (Figure 1A). The presence of these intersecting targets provides a crucial foundation of biological relevance for subsequent network pharmacology analyses, consistent with the field’s recognised multi-dimensional and complexly interacting pathological mechanisms of pruritus [54,55].

### 3.2. PPI Networks of P. odoratum and Levocetirizine with Pruritus Intersection Targets, Respectively

#### 3.2.1. Construction Analysis of PPI Network and Core Target Acquisition

A protein–protein interaction (PPI) network of 113 potential targets was built via the STRING database by inputting the overlapping targets of *P. odoratum* components and pruritus-related diseases. This network featured 113 nodes and 157 edges, with an average node degree of 2.78. PPI enrichment analysis delivered a statistically significant *p* value below 1.0 × 10^−16^, reflecting highly significant interactions among these targets (Figure 1B). Likewise, a PPI network for the 26 shared targets of pruritus and levocetirizine was generated, consisting of 25 nodes and 47 edges, and an average node degree of 3.76 (Figure 1C). These results imply that a single compound can influence multiple targets, and one target can be affected by multiple compounds. This underscores the potential synergistic effects of *P. odoratum* fractions in pruritus therapy by modulating multiple targets.

Cytoscape software was utilized to analyze the constituents of *P. odoratum* and the genes associated with the corresponding targets, thereby constructing a “TCM-active ingredient-target” network (Figure 1D). The CytoNCA plugin in Cytoscape software was used to analyze the PPI network targets. The core network from this analysis shows key characteristics of the PPI network that may have specific biological importance. Network analysis of 111 targets showed a median of 3 for betweenness centrality (BC), 0.05750 for nearness centrality (CC), and 3 for degree centrality (DC), which constructed the key network of *P. odoratum* for the treatment of pruritus (Figure 1E). After 2 median screenings, the median of BC was found to be 11.23333, the median of CC was 0.48148, and the median of DC was 5. Finally, 10 core targets, such as HSP90AA1, JUN, FOS, EP300, EGFR, RPS6KB1, AKT2, ESR1, HSP90AB1, and SRC, were screened out (Table 2 and Figure 1F, for details of the two-step screening of CytoNCA, please refer to Supplementary Table S3), which showed high synergistic effects. We applied the same methodology to examine the 138 concatenated targets of *P. odoratum* and levocetirizine for treating itching. The PPI network comprised 137 nodes and 237 edges, with an average node degree of 3.46. The PPI enrichment analysis yielded a highly significant *p* value of less than 1.0 × 10^−16^, indicating a strong enrichment of interactions among these targets. Following median-based screening of the protein–protein-interaction network (n = 137 nodes), the thresholds were set at BC = 14.81, CC = 0.43 and DC = 5, yielding 14 high-centrality seed genes: SRC, IL6, HSP90AA1, JUN, TNF, ESR1, HSP90AB1, EGFR, EP300, CXCL8, FOS, AKT2, ESR2, and INS. Of these, IL6, TNF, CXCL8, ESR2, and INS were selectively associated with the anti-pruritic response to levocetirizine. Collectively, the results establish that *P. odoratum* exerts pluripotent activity against pruritus by modulating a tightly interconnected core of ten signature targets.

#### 3.2.2. MCODE Analysis of PPI Networks of Intersecting Targets and Functional Enrichment Analysis of the Obtained Clusters

**Figure 2:**
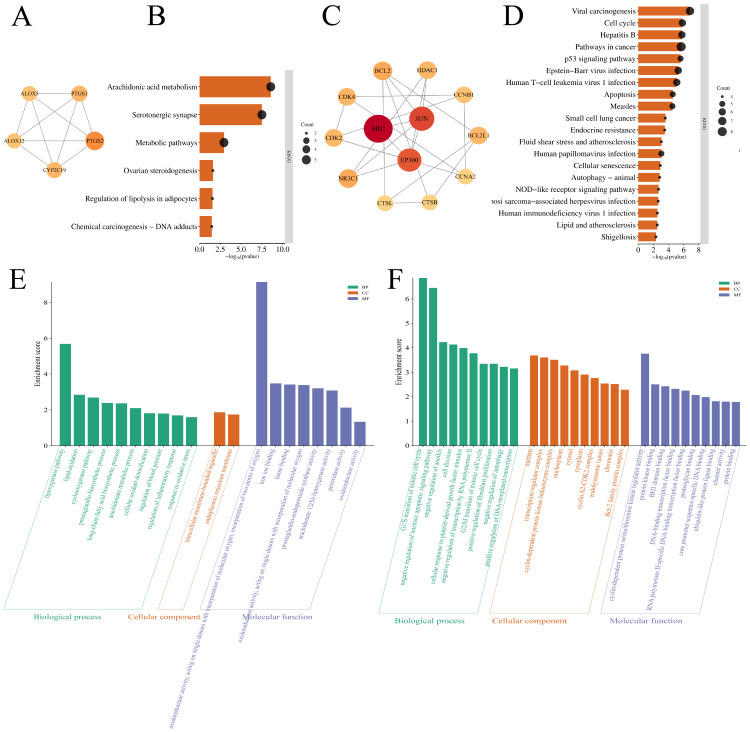
GO and KEGG analysis of the PPI network module for the analysis of core targets by the MCODE plugin. (**A**) MCODE plugin analysis of the top 1 clustered target clusters. (**B**) MCODE plugin analysis of KEGG analysis of the top 1 clustered targets. (**C**) MCODE plugin analysis of the top 2 clustered target clusters. (**D**) MCODE plugin analysis of KEGG analysis of the top 2 clustered targets. (**E**) MCODE plugin analysis of the top 1 clustered targets for GO analysis, green bars represent biological processes (BPs), orange bars represent cellular components (CCs), and blue bars represent molecular functions (MFs), and later the same. (**F**) MCODE plugin analysis of the GO analysis of the clustered targets ranked.

MCODE, a widely used algorithm for mining protein complexes, was employed to perform protein clustering and construct functional modules. The analysis using the MCODE v2.0.3, a plugin, yielded four groups of clustering results (Table 3 and Table S4). The first two clusters were selected for detailed analysis (Figure 2). Cluster 1 contained five targets (Figure 2A) and 20 Gene Ontology (GO) terms (Figure 2E), including 10 BPs, 2 CCs, and 8 MFs, with 6 KEGG pathways identified (Figure 2B). Cluster 2 included 13 targets (Figure 2C) and 86 GO terms (Figure 2F), with 11 BPs, 1 CC, 6 MFs, and 5 KEGG pathways (Figure 2D). The targets contained in cluster 1 involved pathways such as the arachidonic acid metabolism, serotonergic synapse, and ovarian steroidogenesis, etc. The pathways involved in the targets in cluster 2 are the cell cycle, p53 signaling pathway, and NOD-like receptor signaling pathway, etc. GO-BP analysis of the two clusters separately revealed that cluster 1, represented by the PTGS2, is involved in the lipoxygenase pathway, cyclooxygenase pathway, prostaglandin biosynthetic process, and arachidonic acid metabolic process, etc. Cluster 2, represented by the SRC, is involved in the positive regulation of fibroblast proliferation, cellular response to platelet-derived growth factor stimulus, etc.

### 3.3. GO and KEGG Enrichment Analysis of P. odoratum and Levocetirizine with Pruritic Intersection Targets

**Figure 3:**
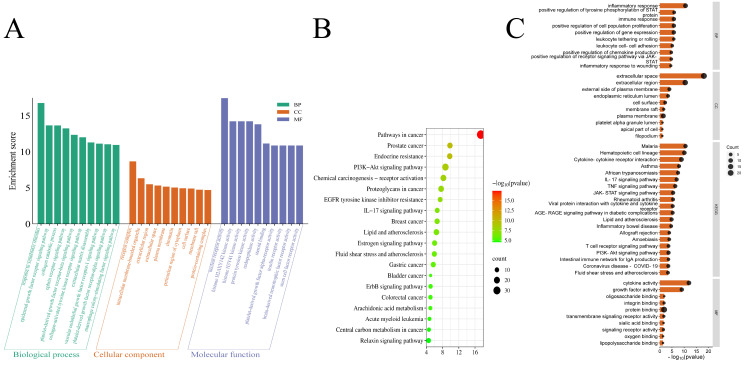
GO and KEGG analysis results of *P. odoratum* and levocetirizine interacting with pruritus, respectively. (**A**) GO analysis of targets interacting with pruritus by *P. odoratum*. (**B**) KEGG analysis of targets interacting with pruritus by *P. odoratum*. (**C**) GO and KEGG analysis of targets interacting with pruritus by levocetirizine.

**Figure 4:**
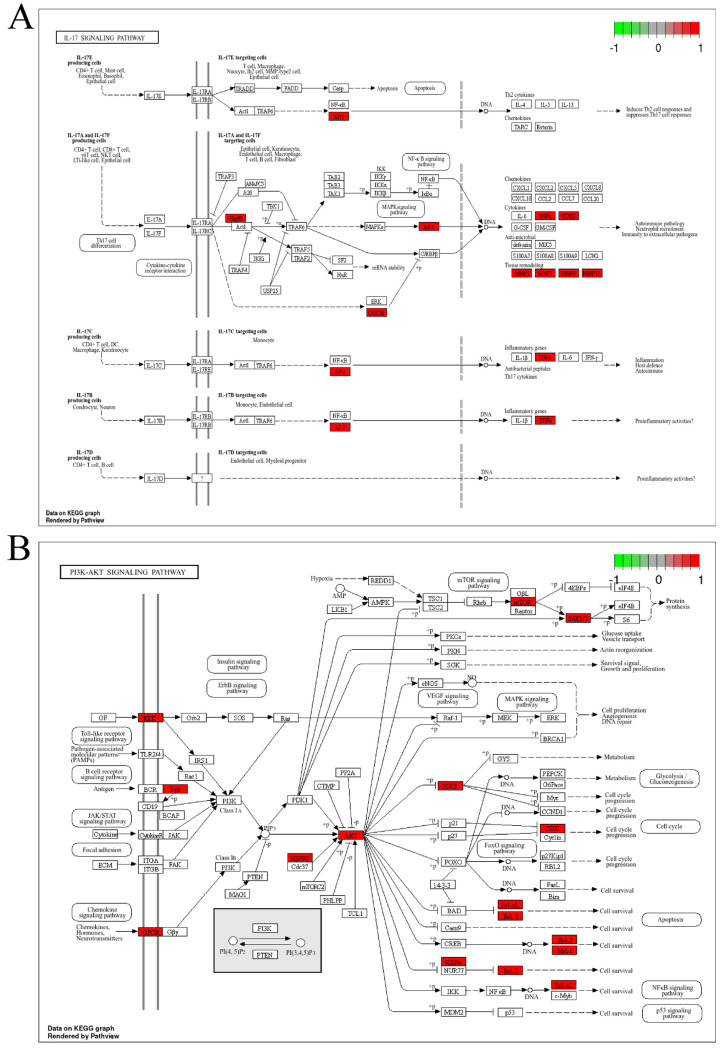
Diagram of IL-17 signaling pathway and PI3K/Akt signaling pathway of KEGG analysis, the target of intersection between *P. odoratum* and pruritus. (**A**) *P. odoratum* interacts with pruritus target KEGG to analyze the cancer pathway of potential targets, and (**B**) *P. odoratum* interacts with pruritus target KEGG to analyze the PI3K/Akt signaling pathway of potential targets. The red part indicates the target of *P. odoratum*’s action.

The Gene Ontology (GO) enrichment analysis of the overlapping targets of *P. odoratum* with pruritus revealed 308 Biological Process (BP) terms, 52 Cellular Component (CC) terms, and 156 Molecular Function (MF) terms (Supplementary Table S5). The top 10 BP entries included response to xenobiotic stimulus, epidermal growth factor receptor signaling pathway, collagen catabolic process, ephrin receptor signaling pathway, and extracellular matrix (ECM) disassembly, etc. The top 10 CC entries were primarily related to receptor complexes, extracellular regions, extracellular space, and intracellular membrane-bound organelles. The top 10 MF entries were mainly linked to protein tyrosine kinase activity, endopeptidase activity, brain-derived neurotrophic factor receptor activity, steroid binding, and nuclear receptor activity, etc. These results are illustrated in Figure 3A. The aforementioned GO terms primarily participate in itch-related signal recognition and transmission (e.g., receptor complexes, kinase activity, signaling pathways), production and action of inflammatory mediators (e.g., extracellular region, endopeptidase activity), neuro-immune cross-regulation (e.g., membrane raft, brain-derived neurotrophic factor receptor activity), as well as nuclear receptor-mediated transcriptional regulation and cellular structural dynamics. These constitute the key molecular and cellular basis for the development and progression of pruritus. The intersection targets were also analyzed through KEGG pathway enrichment, identifying 108 signaling pathways (Supplementary Table S5). The top 20 pathways were screened in ascending order of *p* value, mainly involving the PI3K/Akt signaling pathway, the IL-17 signaling pathway, arachidonic acid metabolism, etc. (Figure 3B), which were further visualized in KEGG by pathway maps, and the IL-17 signaling pathway (Figure 4A). In addition, the PI3K/Akt signaling pathway is an inflammation-related signaling pathway (Figure 4B). In addition, KEGG pathway enrichment analysis identified the top 20 pathways, among which the IL-17 and PI3K/Akt signaling pathway were prominent. Other notable signaling pathways included the estrogen signaling pathway, ErbB signaling pathway, and relaxin signaling pathway (Figure 3B). These findings suggest that the potential targets of *P. odoratum*’s therapeutic action for pruritus management are distributed across multiple pathways. The observed effects may be attributed to synergistic modulation of multiple structures or functions, or they may be the result of complex, multi-pathway intersections.

Subsequently, the core genes involved in the action of levocetirizine on pruritus were analyzed and compared with the corresponding GO and KEGG pathways (Figure 3C and Supplementary Table S6). The results revealed that levocetirizine primarily participates in the following pathways: IL-17 signaling pathway, T cell receptor signaling pathway, and other immune-related pathways. It was also implicated in signaling pathways, including the TNF signaling pathway, JAK/STAT signaling pathway, and PI3K/Akt signaling pathway. Furthermore, we analyzed the core targets of pruritus shared between *P. odoratum* and levocetirizine and examined their associated GO and KEGG pathways. The findings revealed that the PI3K/Akt signaling pathway and the IL-17 signaling pathway are important shared pathways for pruritus treatment. However, there are also differences in the pathways involved in pruritus management. For instance, levocetirizine-specific pathways for pruritus treatment include those related to inflammatory bowel disease and asthma.

### 3.4. Drug–Component–Target–Pathway Network for the Treatment of Pruritus in P. odoratum

**Figure 5:**
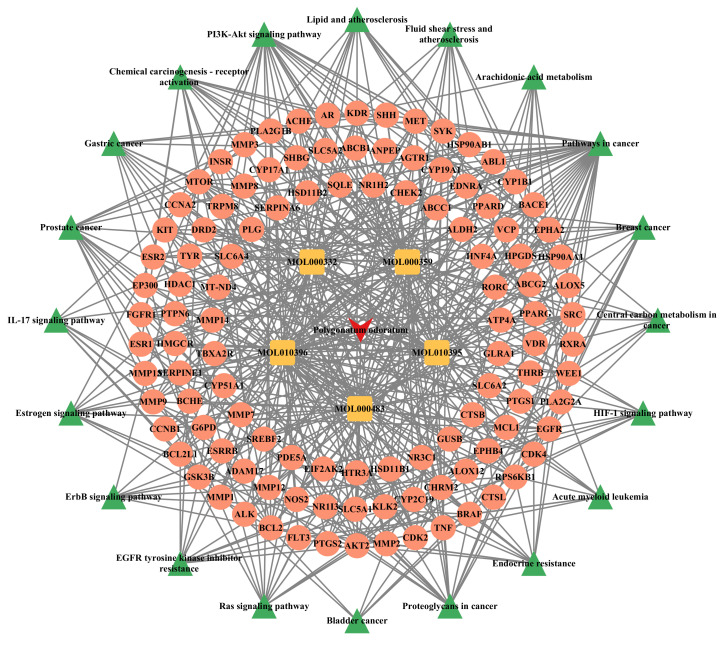
The “drug-component-target-pathway” network of *Polygonatum odoratum* in the treatment of pruritus.

In this network, the average degree value of 113 potential target genes for *P. odoratum* in treating pruritus is 4.87. The top five target genes with the highest degree values are BRAF, RPS6KB1, MTOR, EGFR, and AKT2. Numerous related signaling pathways are involved, such as cancer pathways, prostate cancer, PI3K/Akt signaling pathway, proteoglycans in cancer, endocrine resistance, etc. (Figure 5).

### 3.5. Molecular Docking Validation of P. odoratum Compounds with 10 Core Targets

**Figure 6:**
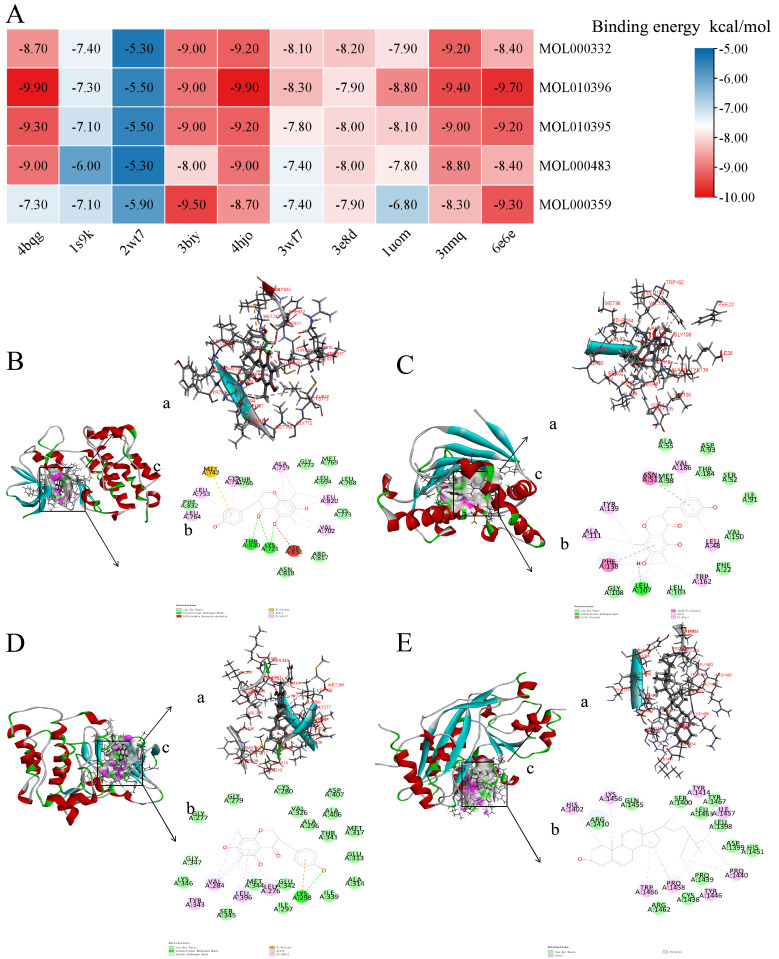
Heat map of molecular docking fractions, and four typical molecular docking diagrams of *P. odoratum* in the treatment of pruritus. (**A**) Heat map of the docking fractions of core targets and highly active small molecules of *P. odoratum*. (**B**) Binding mode of MOL010396 to the 4hjo protein pocket. (**C**) Binding mode of MOL010396 to the 4bqg protein pocket. (**D**) Binding mode of MOL010396 to the 6e6e protein pocket. (**E**) Binding mode of MOL000359 to the 3biy protein pocket. Intersection force (a), 2D binding mode (b), and binding pocket (c).

The 10 core targets, including HSP90AA1 (PDB ID: 4bqg, 1.90 Å), JUN (PDB ID: 1s9k, 3.10 Å), FOS (PDB ID: 2wt7, 2.30 Å), EP300 (PDB ID: 3biy, 1.70 Å), EGFR (PDB ID: 4hjo, 2.75 Å), RPS6KB1 (PDB ID: 3wf7, 1.85 Å), AKT2 (PDB ID: 3e8d, 2.70 Å), ESR1 (PDB ID: 1uom, 2.28 Å), HSP90AB1 (PDB ID: 3nmq, 2.20 Å), and SRC (PDB ID: 6e6e, 2.15 Å), were selected for molecular docking analysis. The docking results, presented as a heatmap, identified 50 protein–ligand pairs with docking scores below −5 kcal/mol, with the highest binding energy reaching −9.90 kcal/mol. This indicates that the active components of *P. odoratum* exhibit strong binding energy with these core targets involved in pruritus signaling pathways (Figure 6A).

Four representative molecular docking diagrams are presented in Figure 6B–E. Figure 6B illustrates the docking of 4hjo with MOL010396. Analysis of noncovalent interactions reveals where the small molecule forms hydrogen bonds with Lys721 and Thr766. Figure 6C shows the interaction between 4bqg and MOL010396, with hydrogen bonds observed between the small molecule and Ser52, as well as Tyr139. Lastly, Figure 6D displays the docking of 6e6e with MOL010396, highlighting hydrogen bonds established with Lys298 and Met344. Figure 6E shows the docking of 3biy with the small molecule MOL000359. Analysis of noncovalent interactions reveals that the small molecule forms a hydrogen bond with ARG1410 on the protein (Table 4).

### 3.6. Structural Stability Obtained from Molecular Dynamics Simulations

**Figure 7:**
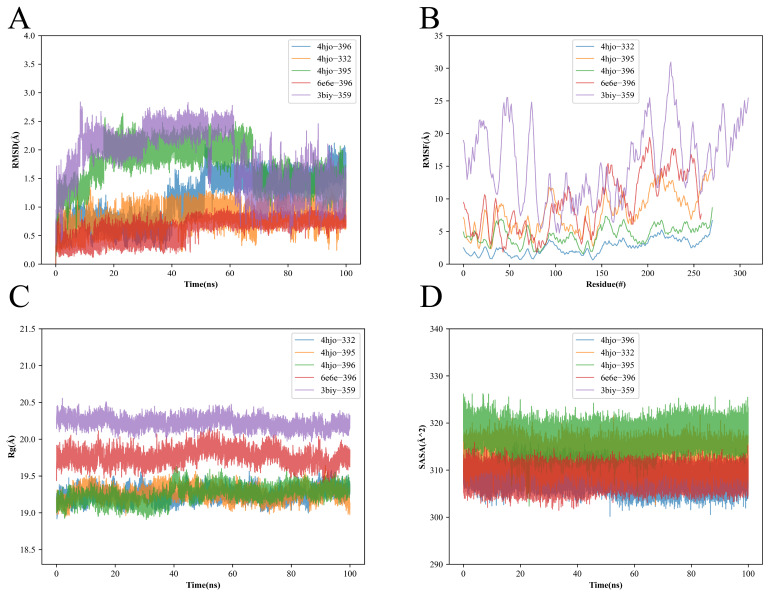
Molecular dynamics simulation study. (**A**) RMSD curves. (**B**) RMSF curves. (**C**) Radius of rotation curves. (**D**) SASA analysis.

After identifying the lowest-energy poses by docking, we selected the five top-scoring compounds for 100 ns molecular dynamics simulations of their complexes with the targets: 4hjo-MOL010396, 4hjo-MOL000332, 4hjo-MOL010395, 6e6e-MOL010396, and 3biy-MOL000359. Binding energies are listed in Table 5, and the resulting conformations together with contacting residues are shown in Figure 7A–D. Based on the MM/PBSA binding free energy values (Table 5; e.g., −18.62 kcal/mol for 4hjo-MOL010396, −23.18 kcal/mol for 4hjo-MOL000332, and −15.74 kcal/mol for 6e6e-MOL010396), a multi-dimensional analysis was performed to characterize the binding stability of the ligand–target complexes.

The Root Mean Square Deviation (RMSD) curves were used to measure the structural changes in the proteins. As shown in Figure 7A, except for the 3biy-MOL000359 complex, the RMSD curves of the other four complexes all reached a relatively stable phase after 60 ns, and the RMSD fluctuation range across the five complexes was between 0.00 and 2.80 Å. This small fluctuation indicates that the overall conformation of the complexes remained relatively stable throughout the 100 ns simulation.

In contrast, the Root Mean Square Fluctuation (RMSF) analysis provides insights into the local flexibility of individual amino acid residues within the protein. As shown in Figure 7B, although the overall structures of most complexes were stable, certain residues in some of the complexes, especially those located in flexible loop regions or near the binding interface, exhibited significant fluctuations, leading to an RMSF value range of 1.00–32.50 Å across the five complexes. Such local flexibility is quite common in protein–ligand complexes and reflects the dynamic nature of the interaction sites.

The Radius of Gyration (RG) is an important indicator of the overall structural compactness of a protein. Figure 7C shows that the radius of rotation of the five complexes remained stable between 18.00 and 20.60 Å. In addition, the Solvent Accessible Surface Area (SASA) was used to characterize the interaction of the proteins with the surrounding water molecules. Figure 7D shows that the SASA of the five complexes did not change significantly during the simulation, which indicates that the binding of ligands has less effect on the protein structure. The results of molecular dynamics (MD) simulations demonstrated that the ligand–target complexes maintained structural stability over the 100 ns simulation period, as evidenced by RMSD and RG values. The predicted binding modes were sustained via key residue interactions, such as hydrogen bonds and hydrophobic interactions. Moreover, MM/PBSA calculations revealed that the binding process was highly favorable from a thermodynamic perspective, with binding free energy values lower than −15 kcal/mol. Collectively, these results validated the prediction of stable binding between the ligands and their respective targets, confirming that the highly bioactive small molecules derived from *Polygonatum odoratum* can stably localize to the active pockets of core target proteins. This study thus provides computational evidence supporting the hypothesis that the bioactive components of *P. odoratum* exert their biological activities through a synergistic multi-target mechanism of action.

### 3.7. GeneMANIA-Based Functional Association Network Analysis of Core Targets of P. odoratum Against Pruritus

**Figure 8:**
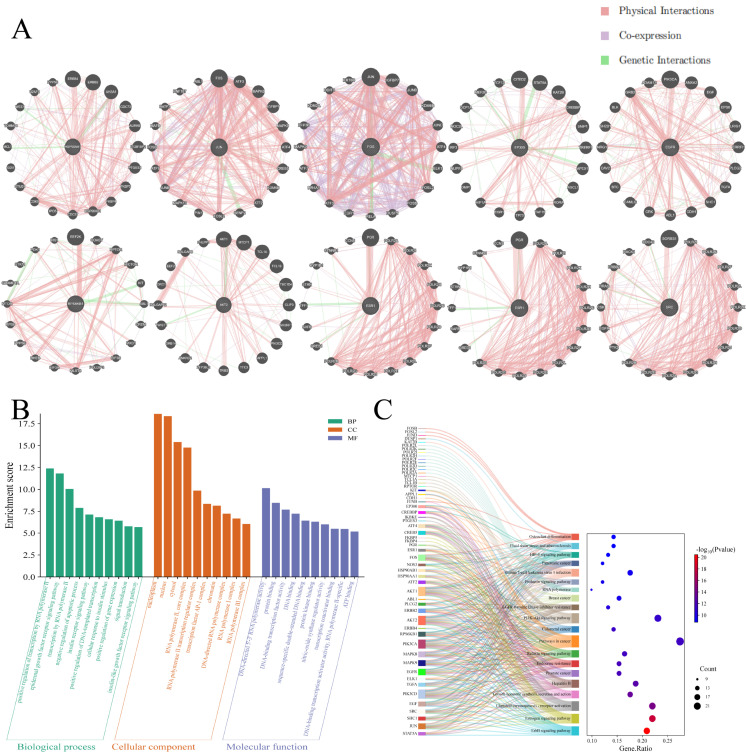
GeneMANIA functional association (GMFA) network with GO, KEGG enrichment analysis of targets identified in the GMFA-ED dataset. (**A**) GeneMANIA functional association (GMFA) network analysis of functionally related genes associated with the first 10 core targets. (**B**) GO analysis of GMFA-ED dataset targets. (**C**) KEGG analysis of GMFA-ED dataset targets.

Using the GeneMANIA analysis method, the GMFA-ED (Supplementary Table S7) was generated by identifying 20 additional genes for each core target (Figure 8A). The top 10 newly identified genes for each core target were then selected to expand the gene list of the top 10 core genes (Supplementary Table S7). After removing duplicates, a total of 91 genes were included. This expanded gene list provides a more comprehensive basis for further analysis.

### 3.8. GO and KEGG Enrichment Analysis of GMFA-ED

We performed GO enrichment analysis on the 91 GMFA-ED targets, obtaining 163 BP, 37 CC, and 130 MF entries. The top 10 BP entries, ranked by ascending *p* value, mainly covered the epidermal growth factor receptor signaling pathway and signal transduction. The top 10 CC entries included nucleus, nucleoplasm, and transcription factor AP-1 complex, while the top 10 MF entries were predominantly DNA-binding transcription factor activity, DNA-binding transcription activator activity, RNA polymerase II-specific, protein binding, and nitric-oxide synthase regulator activity (Figure 8B). KEGG pathway enrichment analysis of the targets identified 128 signaling pathways. The top 20 pathways, ranked by ascending *p* value, primarily included the PI3K/Akt signaling pathway, ErbB signaling pathway, HIF-1 signaling pathway, estrogen signaling pathway, and EGFR tyrosine kinase inhibitor resistance were highlighted (Figure 8C and Supplementary Table S7).

**Figure 9:**
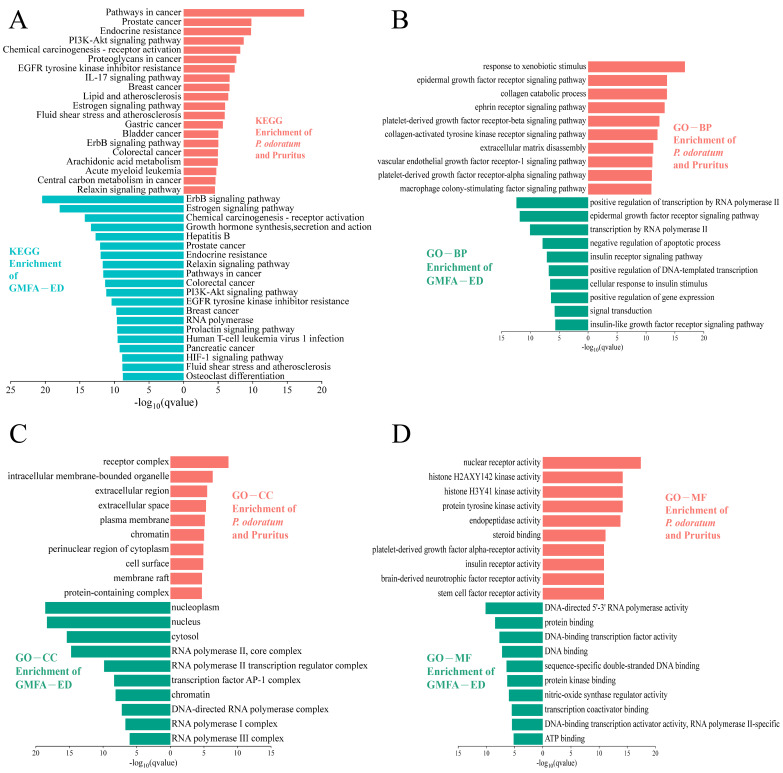
Comparison of KEGG and GO enrichment of intersecting targets of *P. odoratum* on pruritus with GMFA-ED targets. (**A**) KEGG enrichment. (**B**) GO-enriched BP term. (**C**) GO-enriched CC term. (**D**) GO-enriched MF term.

KEGG pathway analysis of targets within GMFA-ED revealed that there was an enrichment of pruritus-related signaling. After GMFA-ED filtering, the ErbB and PI3K/Akt signalling pathways exhibited marked and exclusive enrichment, implicating them as central drivers of the observed phenotype. Figure 9 visually compares the KEGG and GO enrichment analyses conducted before and after the implementation of GMFA (*P. odoratum* and pruritus versus GMFA-ED). The GMFA-ED enrichment analyses offer a comprehensive overview of the enriched BPs, CCs, and MFs linked to *P. odoratum*’s potential targets for combating pruritus. These results show a greater relevance than the initial enrichment of the intersection core targets (*P. odoratum* and pruritus), thus shedding light on how *P. odoratum* might work at the molecular level in pruritus therapy.

**Figure 10:**
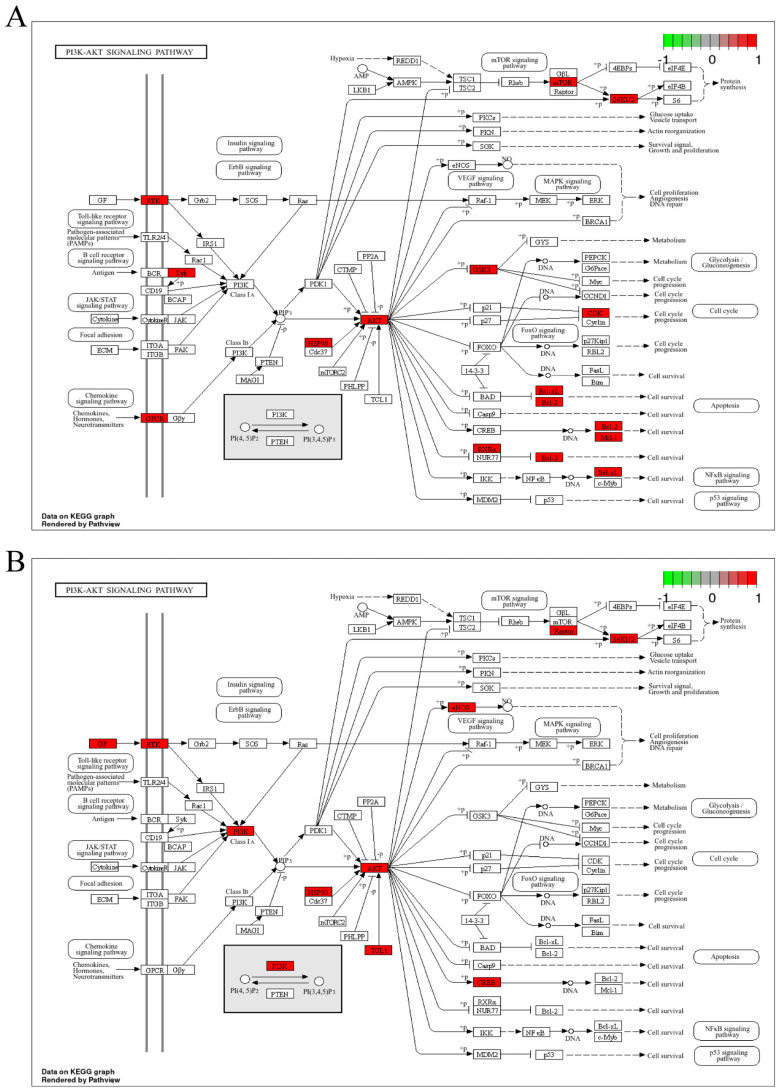
Comparison of KEGG pathway enrichment in pruritus between *P. odoratum* for pruritus and GMFA-ED. (**A**) KEGG analysis of targets of prostate cancer *P. odoratum* interacting with pruritus and (**B**) KEGG analysis of GMFA-ED targets of pruritus after GMFA, with targets significantly enriched in the PI3K/Akt signaling pathway.

After GMFA-ED filtering, KEGG mapping revealed a clear over-representation of the ErbB pathway, where EGF, TGF-α, ErbB1, ErbB2, PI3K, Akt, p70S6K, JNK, JUN, and ELK form one contiguous node. This tight signature was not seen in our earlier *P. odoratum* network, which only sporadically contained FGF, TGF-α, JNK, ELK, and PI3K, demonstrating the selectivity introduced by GMFA-ED weighting (Supplementary Figure S1). Likewise, the screening yielded a focused enrichment within the PI3K/Akt signalling pathway, recruiting GF, RTK, PI3K, AKT, HSP90, CREB, eNOS, and Raptor as a continuous module, whereas the previous network had only RTK, AKT, and HSP90. Thus, the revised weighting protocol reveals a previously hidden branch of the pathway. This discrepancy underscores the potential therapeutic significance of GMFA-ED analysis in elucidating the role of the ErbB and PI3K/Akt signaling pathways in *P. odoratum*-mediated pruritus relief. Moreover, our findings identify several additional candidate therapeutic targets that may be relevant for the treatment of pruritus using *P. odoratum* (Figure 10).

### 3.9. Molecular Docking and Molecular Dynamics Simulation Studies of Pathway Targets

**Figure 11:**
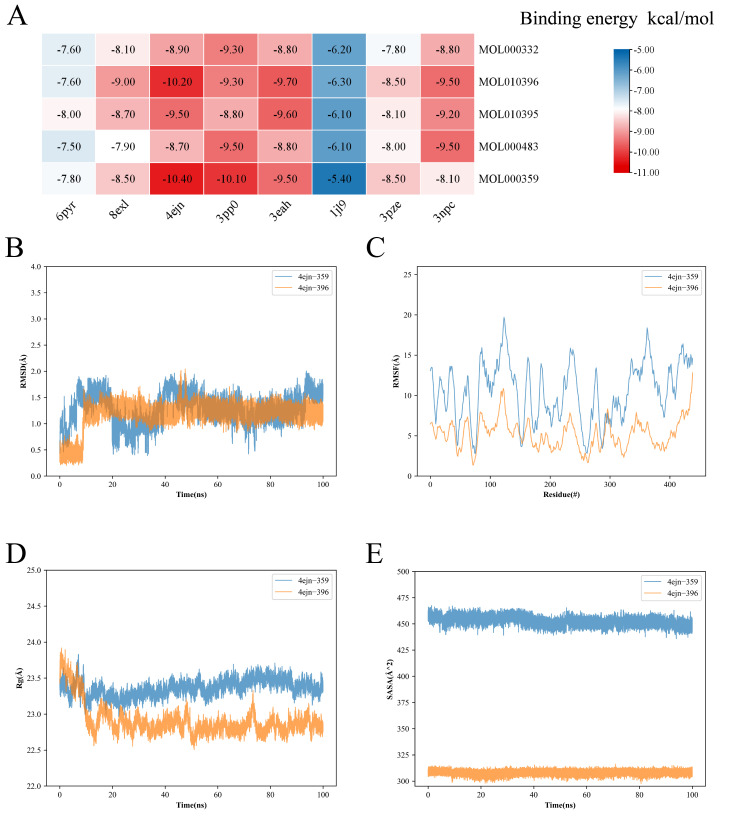
Heatmaps of molecular docking scores and molecular dynamics simulation graphs. (**A**) Molecular docking heat map. (**B**–**E**) Molecular dynamics simulation figure. RMSD curves (**B**). RMSF curves (**C**). Radius of rotation curves (**D**). SASA analysis (**E**).

Interactions between *P. odoratum* and protein targets related to the ErbB signaling pathway and PI3K/Akt pathway were explored by molecular docking and kinetic simulations (Supplementary Table S8). To dissect the ligand–receptor interface underlying the ErbB signaling pathway enrichment, we docked the top-ranked *P. odoratum* metabolites against the catalytic domains of EGF (PDB ID: 1jl9, 3.00 Å) and JNK (MAPK9: 3npc, 2.35 Å; MAPK8: 3pze, 2.00 Å). The lowest-energy complexes converged at −9.5 kcal/mol for 3npc-MOL010396 and 3npc-MOL000483, and −8.5 kcal/mol for 3pze-MOL000359, indicating high-energy engagement of the ErbB signaling pathway by *P. odoratum* constituents (Figure 11A). For the PI3K/AKT pathway, molecular docking was performed with high-activity small molecules derived from *P. odoratum* against the following proteins: PI3K (PIK3CD PDB DI: 6pyr, 2.21 Å and PIK3CA PDB DI: 8exl, 1.99 Å), AKT1 (PDB DI: 4ejn, 2.19 Å), GFR (ERBB2 PDB DI: 3pp0, 2.25 Å), and eNOS (NOS3 PDB DI: 3eah, 2.44 Å). *P. odoratum* had a strong energy for the PI3K/Akt pathway (Figure 11A and Figure S2), and 4ejn-MOL000359 (−10.40 kcal/mol), 4ejn-MOL010396 (−10.20 kcal/mol), 3pp0-MOL000359 (−10.10 kcal/mol), and 3pp0-MOL000483 (−9.50 kcal/mol) had lower binding energies. As part of the analysis of the first 10 core genes identified earlier, molecular docking and MD mimicry of other targets in the PI3K/Akt signaling pathway have been investigated.

Based on the docking results, molecular dynamics (MD) simulations were performed for the first two complexes with lower binding energies and binding activity. The binding free energy data (Table 6) showed that the MM/PBSA binding free energies of the two complexes were −25.21 kcal/mol (4ejn-MOL000359) and −22.33 kcal/mol (4ejn-MOL010396), respectively. Both values were negative and of high absolute magnitude, indicating that the two ligands possess the potential for spontaneous binding to the target in thermodynamic terms. The RMSD analysis showed that the RMSD values of the two complexes were slightly higher (0.20–2.00 Å), but the 4ejn-MOL000359 complex tended to be stabilized after 40 ns, whereas the 4ejn-MOL010396 complex reached a steady state after 10 ns. Thus, as a whole, both complexes showed a relatively stable conformation during the simulation (Figure 11B). RMSF analysis showed that the overall structures of the two complexes were relatively stable, but there were some fluctuations in some regions (2.00–20.00 Å). Among them, the RMSF of the 4ejn-MOL010396 complex was more significant (Figure 11C). This suggests that higher flexibility may exist in these regions, but does not affect the stability of the overall structure. RG analysis showed that the radius of rotation of the protein remained relatively stable throughout the simulation. Although the radius of rotation of the 4ejn-MOL010396 complex deviated transiently at the beginning of the simulation, it quickly regained stability after 15 ns (Figure 11D). SASA analysis showed no significant changes in the two complexes, indicating that the binding of these 2 ligands has less effect on the structure of the 4ejn protein, while the significant difference in the values of the complexes in the figure is related to the different ligands (Figure 11E). In summary, both MOL000359 and MOL010396, identified as active small molecules derived from *P. odoratum*, demonstrated stable binding within the binding pocket of the AKT1 protein (PDB ID: 4ejn). This finding further corroborates the existence of robust interactions between the active constituents of *P. odoratum* and the selected target protein. Moreover, it provides compelling theoretical evidence supporting the pharmacological mechanisms underlying the therapeutic effects of *P. odoratum*’s active ingredients.

### 3.10. Differential Expression Analysis Results of Core Targets for P. odoratum Treatment of Itching Based on GEO Database Validation

**Figure 12:**
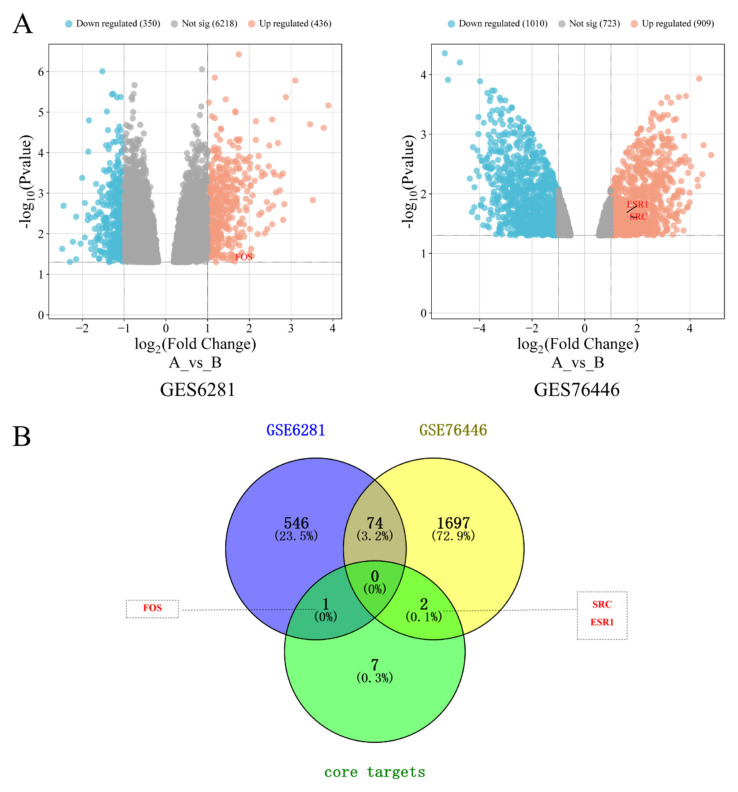
Validation of core targets for *P. odoratum* treatment of pruritus in GEO datasets (GSE6281 and GSE76446). (**A**) Volcano plots for GSE6281 and GSE76446, where A represents the disease group and (**B**) represents the normal group; Venn diagram of GSE6281, GSE76446, and core targets.

Using itch-related datasets (GSE6281 and GSE76446) from the GEO database, differential expression analysis was performed via the GEO2R tool. Screening criteria: |log_2_FC| > 1 and *p* < 0.05 (Supplementary Table S9). The volcano plot (Figure 12A) displays the overall distribution of differentially expressed genes (DEGs). To validate the reliability of network pharmacology predictions, the 10 core targets (HSP90AA1, JUN, FOS, EP300, EGFR, SRC, AKT2, ESR1, HSP90AB1, and RPS6KB1) were cross-analyzed with the aforementioned DEGs (Figure 12B). Results indicated that FOS was upregulated in GSE6281 (log_2_FC = 1.65, *p* < 0.05); SRC and ESR1 were upregulated in GSE76446 (log_2_FC = 1.69, 1.56, *p* < 0.05; small sample size (2 vs 2); results are for reference only and should be interpreted with caution). Given the limited sample size of GSE76446, the above findings are suggestive in nature, and definitive conclusions await validation in larger cohorts or independent clinical samples. These findings suggest that FOS, SRC, and ESR1 represent credible core targets for *P. odoratum*’s treatment of pruritus, providing experimental evidence and directional clues for subsequent mechanistic studies.

## 4. Discussion

This study systematically explored the multi-component, multi-target, and multi-pathway mechanisms underlying the antipruritic effects of *P. odoratum* using an integrated approach combining network pharmacology, molecular docking, molecular dynamics (MD) simulation, GeneMANIA functional association analysis (GMFA), and GEO dataset validation. The findings reveal that *P. odoratum* may exert its therapeutic effects on pruritus through a synergistic mechanism involving the inhibition of inflammation, repair of skin barrier integrity, and modulation of neural signaling pathways, thereby providing a novel perspective for the development of plant-based interventions for chronic pruritus. Network pharmacology analysis identified five major bioactive components in *P. odoratum* that target a total of 208 molecular entities, among which 113 were predicted to be associated with pruritus regulation, and ten were identified as core targets, including HSP90AA1, JUN, FOS, EP300, EGFR, SRC, AKT2, ESR1, HSP90AB1, and RPS6KB1. These targets are functionally interconnected and play pivotal roles in distinct aspects of pruritus pathogenesis. Specifically, HSP90, JUN (AP-1), and FOS are critically involved in amplifying neurogenic inflammation and itch signaling. HSP90, upregulated in TPA-induced skin inflammation, contributes to the maintenance of proinflammatory signaling [56]; JUN regulates the IL-23/IL-33 axis and activates TNF-α/JNK signaling to sustain inflammatory loops [57,58]; and FOS co-localizes with pruriceptive GRPR neurons and is upregulated in chronic dry skin models [59,60]. Together, these components form a core inflammatory amplification module. In parallel, EGFR and SRC are key regulators of keratinocyte differentiation, epidermal barrier function, and COX-2-mediated inflammation. Dysregulation of these targets compromises skin barrier integrity, a hallmark of chronic pruritus [61]. EP300 further supports barrier homeostasis by modulating keratinocyte growth and cytokine expression but may exacerbate inflammation upon overactivation [62,63]. AKT2 is implicated in skin development, wound healing, and age-related barrier dysfunction [64,65,66], while RPS6KB1 drives keratinocyte proliferation in Th17-mediated inflammatory pruritus [67,68,69]. ESR1, downregulated in psoriasis, enhances GABAergic inhibition and reduces sensory neuron hyperexcitability, thereby suppressing abnormal itch perception [70,71,72,73,74]. These findings suggest that *P. odoratum* may restore skin barrier function, suppress inflammatory signaling, and modulate neural hypersensitivity through the coordinated regulation of these targets.

Molecular docking and MD simulation results further support the biological relevance of these predictions. Key active components of *P. odoratum* exhibited strong binding energy (<−5 kcal/mol) to critical targets such as SRC and EGFR, and MD simulations confirmed the formation of stable ligand–protein complexes. SRC is known to disrupt keratinocyte adhesion and initiate the “barrier disruption-itch” cascade via phosphorylation of β-catenin and connexins [75], whereas EGFR influences both barrier repair and itch signaling through modulation of the JAK2/STAT and inflammatory mediator pathways [76,77,78,79,80,81,82,83,84]. These interactions highlight the potential of *P. odoratum* components to directly target molecular switches involved in itch development.

Enrichment analyses using GO and KEGG databases identified several critical pathways associated with the antipruritic effects of *P. odoratum*, prominently including the PI3K/Akt, ErbB (EGFR), and IL-17 signaling pathways. The PI3K/Akt pathway regulates cell survival, inflammation, and immune responses and has been implicated in multiple skin disorders with pruritic manifestations [85,86,87]. The ErbB pathway, particularly through EGFR and HER2, promotes keratinocyte proliferation and the release of pruritogenic cytokines such as IL-31 and TSLP, which directly activate sensory neurons [78,79,88,89]. The IL-17 pathway, driven by Th17 cells, exacerbates allergic and inflammatory skin conditions accompanied by intense itching [90]. These pathway-level insights reinforce the notion that *P. odoratum* modulates both cellular and immune mechanisms underlying pruritus.

Further functional clustering analysis of core targets using the MCODE plugin revealed their primary enrichment in two major functional modules: Arachidonic acid metabolism and serotonergic synapse. Arachidonic acid metabolites have been demonstrated to enhance pruritus responses in chronic kidney disease mouse models. As a key regulatory substance in neurodegenerative diseases, metabolic abnormalities may exacerbate pruritus pathology by releasing inflammatory mediators [91]. Furthermore, serotonergic synapses (i.e., 5-HT neurocircuitry) are not only closely associated with itch intensity, inflammatory burden, and emotional disturbances in atopic dermatitis [92] but also participate in regulating synaptic plasticity within the spinal-thalamic pain-itch pathway, serving as a critical link in central sensitization and the maintenance of chronic itch [93,94,95,96]. Furthermore, GMFA-ED analysis identified numerous Gene Ontology (GO) terms potentially associated with the regulation of pruritus. In addition, several key KEGG signaling pathways were highlighted, including PI3K/Akt, ErbB, HIF-1, and the estrogen signaling pathway, all of which play important roles in the onset and progression of pruritus. Notably, the estrogen signaling pathway was significantly enriched, contributing to skin barrier maintenance by promoting epidermal lipid synthesis, modulating inflammatory cell activity, and influencing the function of GRPR-expressing neurons. These mechanisms may collectively contribute to the observed differences in pruritus sensitivity, particularly in females [64,97,98]. Collectively, these pathways form a multidimensional “neuro-immune-metabolic” network, suggesting that *P. odoratum* may exert systemic anti-itch effects by simultaneously modulating key nodes within this network.

Comparison with the positive control drug levocetirizine revealed that the predicted targets of *P. odoratum* were significantly enriched in the estrogen signaling pathway, ErbB/EGFR pathway, PI3K/Akt pathway, and IL-17 pathway. Its mechanism of action is more diverse, encompassing multiple dimensions including inflammation inhibition (PI3K/Akt/IL-17/ErbB), barrier repair (EGFR/SRC/EP300/RPS6KB1), and neuromodulation (serotonergic synapses/estrogen signaling/arachidonic acid metabolism). This multi-pathway integration may partially compensate for the limited efficacy of single antihistamines. The two agents are expected to achieve superior anti-itch synergistic effects through complementary mechanisms.

To enhance the reliability of the predictions, this study further validated core targets (such as FOS, SRC, and ESR1) using two itch-related transcriptomic datasets: GSE6281 and GSE76446. Results showed significant transcriptional abnormalities in these targets within itch samples, highly consistent with functional predictions from the drug–target–disease network. Specifically, FOS participates in spinal itch circuits and inflammatory responses; SRC regulates EGFR-mediated keratinocyte function and itch signaling; and ESR1 modulates GABAergic inhibition and sensory neuron sensitization. Collectively, these three targets may synergistically mediate *P. odoratum*’s anti-itch effects through interactions with core nodes like HSP90/JUN. This validation strategy, based on public data, further enhances the credibility of the predicted core targets.

In this study, network pharmacology predicted that *P. odoratum* may repair the skin barrier through a multi-target mechanism, and this computational finding is consistent with the conclusions of recent applied research on the bioactive components of *P. odoratum*. For instance, patent technology has demonstrated that a specific lectin derived from *P. odoratum* can alleviate inflammation and promote barrier repair by inhibiting hyaluronidase and elastase [99]. Meanwhile, biological polysaccharides prepared from a combination of *P. odoratum* and *Dioscorea opposita* have been proven to synergistically upregulate the expression of ceramide synthase and hyaluronic acid synthase, thereby enhancing barrier function at the source of synthesis [100]. These findings collectively indicate that the therapeutic potential of *P. odoratum* lies in its dual mechanisms of anti-inflammatory and antipruritic effects and tonifying and consolidating the fundamental functions (i.e., skin barrier repair) [101,102]. This multi-target action characteristic is also highly consistent with the current cutting-edge perspective that chronic pruritus arises from the global dysfunction of the neuro-immune-epidermal unit. A recent review pointed out that in diseases such as atopic dermatitis, keratinocytes, immune cells, and sensory neurons form a core feedback loop of inflammation-pruritus-barrier disruption [103], and the multi-target intervention of *P. odoratum* may potentially act on this complex regulatory network [104].

In conclusion, this study reveals the molecular mechanism underlying the antipruritic effect of *P. odoratum* via the synergistic actions of multi-components, multi-targets, and multi-pathways, with its core lying in the simultaneous suppression of inflammatory cascades, restoration of skin barrier function, and regulation of neural sensitization signals. The identified key targets (e.g., PI3K/Akt, IL-17, EGFR, SRC, ESR1) and pathways provide crucial candidate molecules and a theoretical framework for the development of plant-derived antipruritic agents. Although the initially predicted targets from public databases may include indirectly associated genes, PPI network topological analysis has successfully screened out key signaling hubs directly involved in pruritus perception and transduction. It should be noted that this study is mainly based on computational prediction and bioinformatics analysis without experimental validation. While the predicted results provide valuable insights into understanding the antipruritic effects of *P. odoratum*, its specific mechanisms remain to be further verified through in vitro cell experiments, animal models, and clinical studies. At the in vitro level, human immortalized keratinocytes (HaCaT) and pruritus-related cell models should be first employed to evaluate the effects of core components of *P. odoratum* on the expression of inflammatory factors (e.g., IL-6, IL-8, TNF-α) and skin barrier-related proteins (e.g., filaggrin) within safe concentration ranges, and techniques such as SPR should be used to verify their direct binding to key targets (e.g., HSP90AA1, AKT1). At the animal level, an MC903-induced mouse model of chronic pruritus should be established to comprehensively evaluate the in vivo efficacy of *P. odoratum* through behavioral assessment, histopathological examination, and detection of serum inflammatory and barrier markers, to systematically validate its multi-target mechanism of “anti-inflammation, barrier repair, and neural regulation”. The insufficient sample size of GSE76446 represents a limitation of this study, and future work should validate the observed expression changes in relevant targets in larger patient cohorts. We have also recommended subsequent validation using clinical samples from pruritic patients via qPCR or immunohistochemistry. On this basis, multi-omics analyses such as transcriptomics need to be further integrated to clarify the functional roles of core pathways (PI3K/Akt, IL-17, ErbB) and the pharmacodynamic characteristics of active components. In addition, the potential of the combined application of *P. odoratum* with traditional antihistamines (e.g., levocetirizine) could be explored to provide new experimental evidence for the comprehensive treatment of chronic pruritus. The above validations will systematically clarify the biological functions of key hub targets such as SRC, EGFR, and ESR1, thereby constructing a complete evidence chain from computational prediction to experimental validation and preclinical evaluation, which lays a translational foundation for the application of *P. odoratum* in pruritus-related skin diseases.

## 5. Conclusions

**Figure 13:**
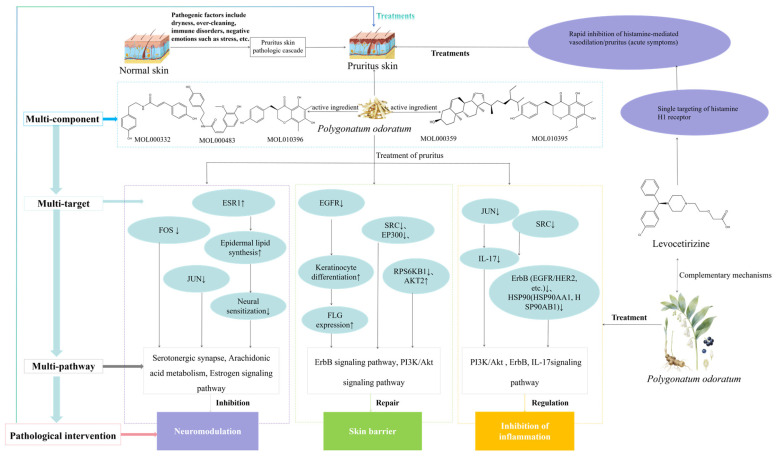
Schematic diagram of the proposed synergistic mechanism of *P. odoratum* in treating itching: “multi-component-multi-target-multi-pathway-pathological intervention”.

This network pharmacology study reveals that *P. odoratum* may treat pruritus through a synergistic mechanism involving inflammation suppression, skin barrier repair, and neuromodulation. Its action targets include key pathways such as PI3K/Akt, IL-17, ErbB (EGFR), and the serotonin/arachidonic acid network. This multidimensional model suggests a potential non-antihistamine natural product therapeutic strategy for chronic pruritus conditions, demonstrating synergistic potential with levocetirizine (Figure 13). This multi-component, multi-target, and multi-pathway synergistic mechanism provides a theoretical basis for developing non-traditional antihistamine natural medicines. It may complement the single antihistamine mechanism of levocetirizine, and combined use could potentially enhance therapeutic efficacy. However, it should be noted that these conclusions are based on bioinformatics and publicly available transcriptomic data. The pharmacological activity, efficacy, and in vivo behavior of the active components still require validation through in vitro and in vivo studies.

## Figures and Tables

**Table 1:** Active ingredients of *P. odoratum*.

Mol ID	Active Ingredient	OB (%)	DL	Pubchem ID
MOL010408	polygosides E_qt	38.73	0.78	N/A
MOL000332	n-coumaroyltyramine *	85.63	0.20	5372945
MOL010396	4’,5,7-trihydroxy-6,8-dimethyl-homoisoflavanone *	59.76	0.30	46886731
MOL010411	4’,5,7-trihydroxy-6-methyl-homoisoflavanone	82.94	0.27	N/A
MOL010395	4’,5,7-trihydroxy-6-methyl-8-methoxy-homoisoflavanone *	89.70	0.33	46886730
MOL010412	4’-methoxy-5,7-dihydroxy-6,8-dimethyl-homoisflavanone	57.14	0.34	N/A
MOL010387	3-o-beta-d-glucopyranosyl-(1-2)-[beta-d-xylopyranosyl-(1-3)]-beta-d-glucopyranosyl-(1-4)-galactopyranosyl-25(S)-spirost-5(6)-en-3beta,14alpho-diol_qt	104.58	0.79	N/A
MOL000483	(Z)-3-(4-hydroxy-3-methoxy-phenyl)-N-[2-(4-hydroxyphenyl)ethyl]acrylamide *	118.35	0.26	6440659
MOL000359	β-sitosterol *	36.91	0.75	12303645

“*” indicates that *P. odoratum* has components that correspond to the target.

**Table 2:** The core targets of the PPI network of *P. odoratum* compounds and disease intersection targets.

Protein Name	Betweenness	Closeness	Degree
HSP90AA1	97.01789322	0.619047619	12
JUN	102.6261905	0.604651163	11
FOS	14.96666667	0.541666667	8
EP300	106.4388889	0.565217391	9
EGFR	13.37777778	0.509803922	6
RPS6KB1	11.23333333	0.481481481	6
AKT2	45.45649351	0.52	8
ESR1	27.84141414	0.590909091	10
HSP90AB1	72.95036075	0.604651163	11
SRC	176.8699856	0.65	13

**Table 3:** MCODE analysis of the intersection targets of the active ingredient (*P. odoratum*) and disease (pruritus).

Cluster	Value	Nodes	Edges	Targets ID
1	5.00	5	10	ALOX5, ALOX12, CYP2C19, PTGS2, PTGS1
2	3.67	13	22	SRC, EP300, JUN, NR3C1, BCL2, BCL2L1, CDK4, CDK2, HDAC1, CCNB1, CCNA2, CTSB, CTSL
3	3.33	4	5	SQLE, CYP51A1, HMGCR, SREBF2
4	3.00	7	9	FOS, ESR2, AR, EGFR, SP90AB, SP90AA, RPS6KB1

**Table 4:** Summary of four sets of typical molecular docking scores and key interactions.

Protein	PBD ID	Ligand	Binding Energy (kcal/mol)	Hydrophobic Interaction	Hydrogen Bond
EGFR	4hjo	MOL010396	−9.90	Val702, Ala719, Lys721, Leu753, Thr766, Leu820	Lys721, Thr766
HSP90AA1	4bqg	MOL010396	−9.90	Leu48, Asn51, Leu107, Phe138, Val186	Ser52, Tyr139
SRC	6e6e	MOL010396	−9.70	Leu276, Ala296, Lys298, Val326, Thr341, Leu396	Lys298, Met344
EP300	3biy	MOL000359	−9.50	LEU1398, HIS1402, TYR1414, PRO1439, PRO1440, GLN1455, ILE1457, LEU1463, TRP1466	ARG1410

**Table 5:** Binding energies of the five complexes.

Protein-Component	Binding Energy (kcal/mol)	Std. Dev.	Std. Err. of Mean
4hjo-396	−18.62	2.72	0.61
4hjo-332	−23.18	4.19	0.94
4hjo-395	−17.76	3.05	0.68
6e6e-396	−15.75	5.86	1.31
3biy-359	−29.6473	3.3756	0.7548

**Table 6:** Binding energies of the 2 complexes.

Protein-Component	Binding Energy (kcal/mol)	Std. Dev.	Std. Err. of Mean
4ejn-359	−25.21	5.39	1.20
4ejn-396	−22.33	3.69	0.82

## Data Availability

The authors confirm that the data supporting the findings of this study are available within the article and its Supplementary Materials.

## Supplementary Materials

The following supporting information can be downloaded at https://www.mdpi.com/article/10.3390/cimb48040369/s1, Supplementary Tables (Table S1: GridBox settings for proteins; Table S2: Information on potential targets of 208 *P. odoratum* active compounds; Table S3: cytoNCA screening for key targets; Table S4: GO functional terms and KEGG enrichment analysis results for the top 2 MCODE network clusters; Table S5: GO functional terms and KEGG enrichment analysis results for 113 potential targets; Table S6: GO functional terms and KEGG enrichment analysis results for 26 targets of levocetirizine acting on pruritus; Table S7: GeneMANIA analysis and GMFA-ED corresponding GO functional terms and KEGG enrichment analysis results; Table S8: Summary of molecular docking scores between *P. odoratum* targets and those associated with the ErbB signaling pathway and PI3K/AKT pathway; Table S9: Summary table of differentially expressed genes identified after performing differential analysis on GSE6281 and GSE76446 using the GEO2R tool); Supplementary Figures (Figure S1: Comparison of KEGG pathway enrichment for pruritus and GMFA-ED in *P. odoratum*. (A) KEGG analysis of *P. odoratum* interaction targets intersecting with pruritus. (B) KEGG analysis of pruritus GMFA-ED targets post-GMFA, with significant enrichment in the ErbB signaling pathway; Figure S2: Molecular docking of 4ejn with MOL000359 and MOL010396, and 3pp0 with MOL000359 and MOL000483. (A) Binding mode of MOL000359 with the 4ejn protein pocket. (B) Binding mode of MOL010396 with the 4ejn protein pocket. (C) Binding mode of MOL000359 with the 3pp0 protein pocket. (D) Binding mode of MOL000483 with the 3pp0 protein pocket. Interaction forces (a), 2D binding mode (b), and binding pocket (c)).

## Abbreviations

The following abbreviations are used in this manuscript:

MD	Molecular dynamics
GMFA	GeneMANIA-based functional association analysis
GO	Gene ontology
KEGG	Kyoto Encyclopedia of Genes and Genomes
*P. odoratum*	*Polygonatum odoratum* (Mill.) Druce
TCMSP	Traditional Chinese Medicine Systems Pharmacology Database and Analysis Platform
OB	Oral bioavailability
DL	Drug likeness
TTD	Therapeutic Target Database
PPI	Protein–protein interaction
BP	Biological process
CC	Cellular component
MF	Molecular function
RESP	Restrained Electrostatic Potential
AM1-BCC	Austin Model 1 with Bond Charge Correction
.top	topology
.crd	coordinat
GMFA-ED	GMFA-extended database
RMSD	Root Mean Square Deviation
RMSF	Root Mean Square Fluctuation
RG	Radius of Gyration
SASA	Solvent Accessible Surface Area
GEO	Gene Expression Omnibus
AR	Androgen Receptor
5-HT	5-Hydroxytryptamine
