# Supplementary figures and images for: Elucidating the Multi-Target Anti-Pruritic Mechanism of Polygonatum odoratum via Integrated Network Pharmacology, Molecular Simulations, and GEO Dataset Validation
Source: Curr Issues Mol Biol. 2026 Apr 1;48(4):369. doi: 10.3390/cimb48040369 (PMC13115042; doi:10.3390/cimb48040369)

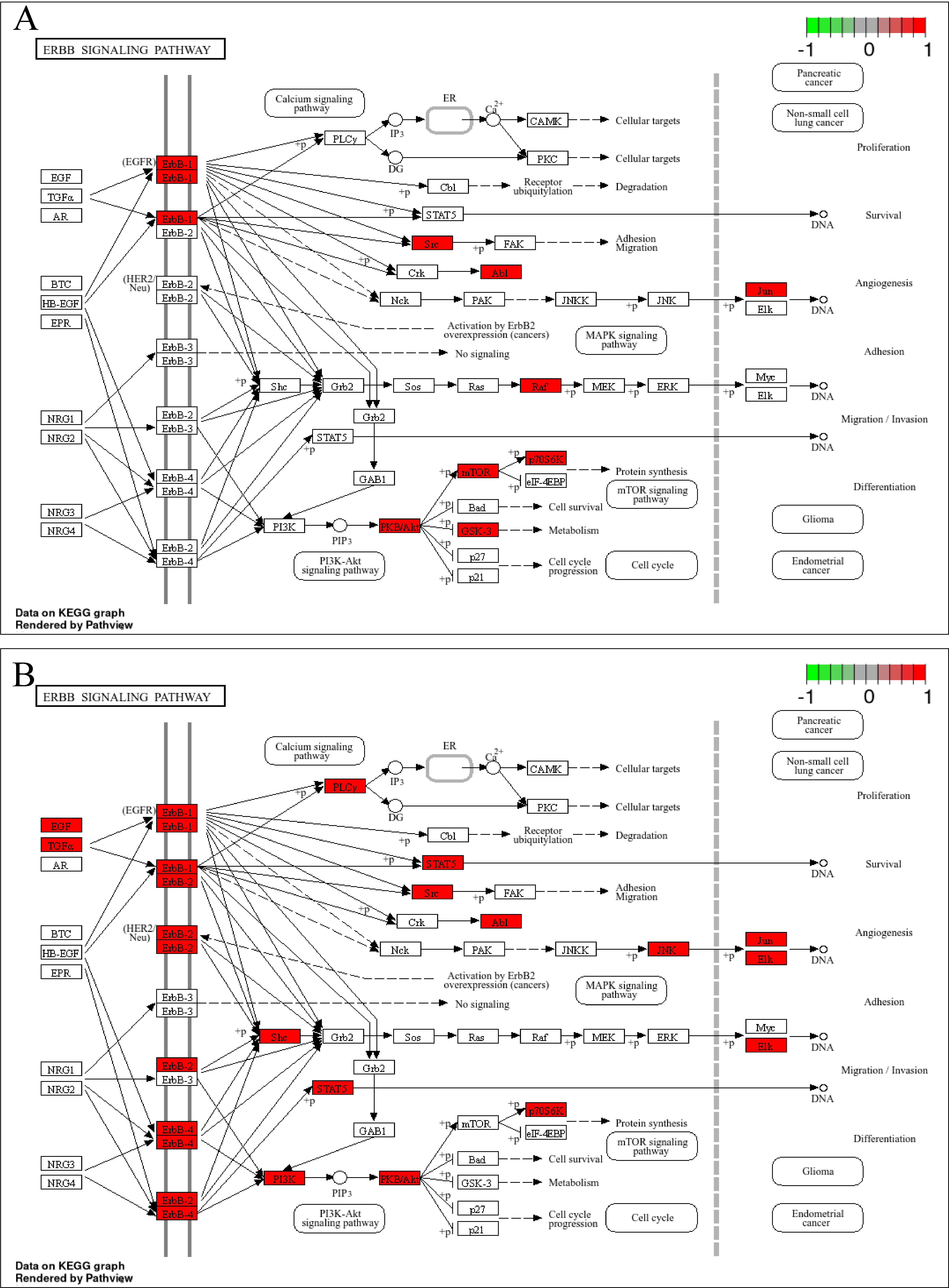

Supplement: Supplementary file 1 [file cimb-48-00369-s001.zip › Supplementary Figures S1. (A,B) Comparison of KEGG pathway enrichment for pruritus and GMFA-ED in P. odoratum.tif]

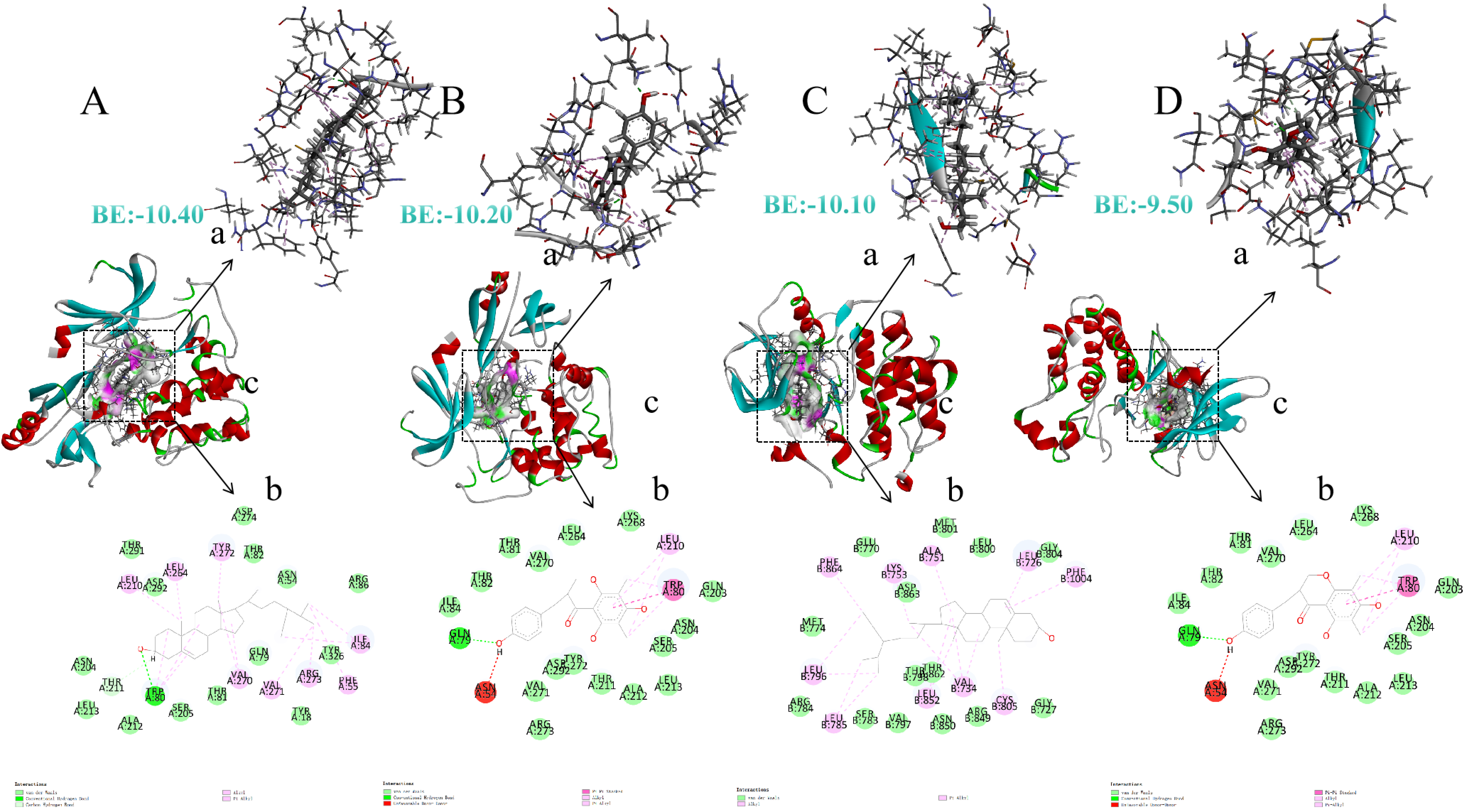

Supplement: Supplementary file 1 [file cimb-48-00369-s001.zip › Supplementary Figures S2. (A-D) Molecular docking diagrams of MOL010396 with 4drj, 4hjo, 4bqg, and 6e6e proteins, respectively.tif]
